# Gait asymmetry, ankle spasticity, and depression as independent predictors of falls in ambulatory stroke patients

**DOI:** 10.1371/journal.pone.0177136

**Published:** 2017-05-23

**Authors:** Ta-Sen Wei, Peng-Ta Liu, Liang-Wey Chang, Sen-Yung Liu

**Affiliations:** 1 Institute of Biomedical Engineering, Colleges of Engineering and Medicine, National Taiwan University, Taipei, Taiwan; 2 Department of Physical Medicine and Rehabilitation & Fall Prevention Center, Changhua Christian Hospital, Changhua, Taiwan; University of Palermo, ITALY

## Abstract

**Background:**

Falls are the leading cause of injury in stroke patients. However, the cause of a fall is complicated, and several types of risk factors are involved. Therefore, a comprehensive model to predict falls with high sensitivity and specificity is needed.

**Methods:**

This study was a prospective study of 112 inpatients in a rehabilitation ward with follow-up interviews in patients’ homes. Evaluations were performed 1 month after stroke and included the following factors: (1) status of cognition, depression, fear of fall and limb spasticity; (2) functional assessments [walking velocity and the Functional Independence Measure (FIM)]; and (3) objective, computerized gait and balance analyses. The outcome variable was the number of accidental falls during the 6-month follow-up period after baseline measurements.

**Results:**

The non-faller group exhibited significantly better walking velocity and FIM scale compared to the faller group (P < .001). The faller group exhibited higher levels of spasticity in the affected limbs, asymmetry of gait parameters in single support (P < .001), double support (P = .027), and step time (P = .003), and lower stability of center of gravity in the medial-lateral direction (P = .008). Psychological assessments revealed that the faller group exhibited more severe depression and lower confidence without falling. A multivariate logistic regression model identified three independent predictors of falls with high sensitivity (82.6%) and specificity (86.5%): the asymmetry ratio of single support [adjusted odds ratio, aOR = 2.2, 95% CI (1.2–3.8)], the level of spasticity in the gastrocnemius [aOR = 3.2 (1.4–7.3)], and the degree of depression [aOR = 1.4 (1.2–1.8)].

**Conclusions:**

This study revealed depression, in additional to gait asymmetry and spasticity, as another independent factor for predicting falls. These results suggest that appropriate gait training, reduction of ankle spasticity, and aggressive management of depression may be critical to prevent falls in stroke patients.

## Introduction

A fall is the common injury in stroke patients. Fall occurrence in stroke survivors is 25–37% within 6 months and 23–50% 6 months post-stroke [1–6]. Accidental falls and fall-related injuries, such as hip fracture, often lead to serious disability and affect the patient’s overall health. Many studies have attempted to identify fall risk factors as predictors and established a sensitive prediction model for stroke patients. Therefore, early interventions for preventing falls may be beneficial to stroke patients.

The causes of fall are complicated, and several factors may result in falls, including impaired balance and gait, declining cognition, muscle weakness, and presence of neurological diseases. Previous studies have demonstrated that balance, walking ability, and physical performance assessments are useful predictors of fall occurrence in stroke patients post-discharge from rehabilitation units [7–9]. These studies have demonstrated that physical performance assessments, including asymmetrical gait pattern, Berg Balance Score (score ≦ 29 at admission), Fall Efficacy Scale (score ≥ 33), and spasticity, predicted the risk of fall in stroke patients to a certain accuracy [10–13].

These findings also suggest that existing predictors of falls exhibit some limitations, especially gait and balance assessments. For example, clinical measures typically assign numerical values to determine the level of performance on tests (e.g., Berg Balance Scale, Performance-Oriented Mobility Assessment, and Dynamic Gait Index). These measurements depend on expert ratings and subjective judgments, and the tests are mostly skill orientated without direct connection to the physiological mechanisms of temporal and spatial characteristics. Therefore, quantified assessments have been developed, and these measurements are more objective than the measurements mentioned above.

A previous study associated impaired balance and gait to increased risk of falls in stroke survivors using quantified measurements [14]. However, the models used for this study did not provide high sensitivity or specificity. Another study also demonstrated that gait and postural variability predicted accidental falls in nursing home residents [10]. The Interactive Balance System correlated with physiological mechanisms of fall, but the predictive ability in this study was limited [10].

Psychological factors may also play an important role in fall occurrence in stroke patients. This concept was supported by results that impaired balance and gait negatively affected psychological distress in stroke survivors [15]. Another study also demonstrated that 30% of stroke patients suffered depression in the early- or late-stage post-stroke [16]. Depression was also a risk related to falls in stroke patients in a previous study [17].

No comprehensive analyses integrate the identified fall risk factors. Quantified gait and balance measurements are more objective and should be used for clinical evaluations. Psychological factors may also be important risk factors for predicting falls in stroke patients. However, studies of fall prediction using objective, quantified gait and balance assessments and psychological evaluations after stroke are limited. Therefore, a prediction model for falls in stroke patients should be developed using a multidimensional assessment to increase prediction accuracy. The present study used physical assessments, including objective computerized gait and balance measurements, and psychological evaluation to identify risk factors related to falls in stroke patients after discharge from hospital and develop a fall prediction model with high sensitivity and specificity.

## Materials and methods

The Institutional Review Board of a tertiary medical center, Changhua Christian Hospital, approved this prospective cohort study, which was performed in a rehabilitation ward and patients’ homes.

### Participants

A total of 140 hospitalized patients who suffered their first stroke were enrolled according to the following criteria: (1) stroke confirmed on MRI or CT; (2) ability to walk independently (with/without assistive device) at least 10 meters; (3) no fall history within 1 year before stroke onset; and (4) written informed consent.

Only patients who met the above entry criteria were included to specifically identify the fall risk factors related to stroke.

A ten-meter walk test was included because it is a valid, reliable assessment for predicting falls in subjects with stroke[12,18]. This test collected dynamic gait parameters as predictors of fall. Therefore, subjects who walked with a person’s assistance had an external supportive force that may interfere with the assessment of gait, and these patients were not included in the present study.

No fall history within 1 year before study entry was selected because subjects who experienced falls may exhibit subsequent factors while walking independently, including fear of a fall, decreased mobility, and changes in gait pattern. Subjects with a history of falling previously may also have their movements closely monitored by their family members or caregivers [12]. These conditions may confound the relationship between variables and fall risk assessment.

Subjects dropped out during the follow-up for the following reasons: nursing home residency (11 subjects), unstable internal disease (8 subjects), unable to complete the interview due to dementia or severe cognitive impairment (4 subjects), loss of contact due to residence address changes (3 subjects), and epilepsy (2 subjects). Therefore, 112 subjects completed the study, and these subjects were further divided into two groups, faller or non-faller, depending on whether a fall occurred during the study period.

### Baseline measurements

Initial assessments were performed at baseline including demographic data and a standardized recording of history and clinical examinations. Baseline physiological and psychological assessments were performed before subjects were discharged from the hospital (approximately 1 month after stroke). These baseline measurements of physical and psychological parameters were used as fall risk factors to develop the fall risk prediction model.

Physical assessments included the Modified Ashworth Scale (MAS), which was used to assess muscle tone in the elbow flexor, knee extensor, and ankle plantar flexor [19]. The level of a patient’s disability was assessed according to the Functional Independence Measure (FIM) [20]. Performance of activities in daily life was assessed during hospitalization according to eighteen items, including bathing, dressing, toileting, transferring, urinary continence, cognitive comprehension and social interaction. This assessment is widely used to measure and predict outcome [21].

The objective measurements of gait were completed using computerized systems with wearable inertial sensors. Subjects wore customized shoes (Ultraflex, Infotronic, the Netherlands) with eight load sensors in each shoe to measure the forces under the foot and detect temporal events in the gait cycle prior to the gait measurements. Data were sampled at the rate of 100 Hz and stored in a portable (Walkman size), lightweight data logger that was carried on the lower back of each subject. Several practice tests were performed before actual data were collected. Subjects walked at a self-selected speed over a 10-m hallway. The mean values of the two tests were used. Gait parameters were normalized to the subject’s body height to account for possible effects of anthropometrics [22,23]. The temporal asymmetry ratios (ASY) for single support time (ASY_ss), double support time (ASY_ds), single swing time (ASY_swing), stance time (ASY_stance), and step time (ASY_step) were quantified using the following equation [24]:

$$\mathrm{Asymmetry}\;\mathrm{ratio}=|1-\frac{\mathrm{affected}\;\mathrm{side}}{\mathrm{unaffected}\;\mathrm{side}}|.$$

A greater value of this ratio indicates higher asymmetry between the two sides.

The objective computerized measurements of balance were completed while the subjects stood on a Stabilo-platform (Ultralfex, Infotronic, the Netherlands) in a comfortable position without footwear or ankle foot orthoses. Subjects kept their eyes open and arms at their sides and were instructed to maintain their balance for 20 seconds [25,26]. Three tests were performed with a 30-second rest between tests. The mean value of three tests is presented. Subjects’ performances were recorded as the center of pressure (COP) trajectory paths. Data were sampled at the rate of 100 Hz, and COP stability was calculated as the standard deviation of the anterioposterior (COP_ap) and mediolateral (COP_ml) directions of the points obtained during measurement. The sway area (COP_area) was calculated as the square root of the sum of squares of the COP_ap and COP_ml.

Psychological evaluations included the Mini-Mental State Examination (MMSE) [27] and Chinese translated version of the Geriatric Depression Scale (GDS) [28], which were used to screen for cognition and depression, respectively. The modified Falls Efficacy Scale (mFES) was used to evaluate the fear of falling in stroke patients [29].

### Assessment of falls

Falls were defined as incidents when the subject came to rest on the floor due to an unexpected loss of balance. All subjects were followed up for 6 months after the first assessment to collect the record of falls. Trained research nurses visited the subjects at home 4, 12, and 24 weeks after discharge from the hospital or rehabilitation ward. Phone reports from subjects were also encouraged in this study to prevent errors from retrospective data collection.

### Statistics

Descriptive analysis was used for all variables, and results are presented as the means, standard deviations and percentages. Significant differences between fallers and non-fallers were assessed using independent Student’s t-test for continuous variables and χ^2^ test analysis for categorical variables. The Mann-Whitney U test was used to detect mean differences between groups when variable distributions were not normal. Linear correlations between continuous variables were calculated using Pearson’s correlation test. Multivariate logistic regression (MLR) analysis was performed using a forward stepwise method with an entry criteria of P = 0.1 to identify the factors that were independently associated with falls. Two models were developed based on variables with statistical significance from bivariate analysis and clinical interests. Adjusted Odds Ratios (aOR) were acquired from the estimated coefficients and presented with the corresponding 95% confidence interval (CI) of the ratio.

The predictive accuracy of the model in discriminating fallers and non-fallers was assessed using sensitivity and specificity. The optimal cutoff point with the highest sensitivity and specificity for each model was defined as the Youden index [30,31].

A receiver operating characteristics (ROC) curve was plotted to assess the discrimination of the generated multivariate logistic models. The area under the curve (AUC) of the ROC was also calculated for each model to determine the fitness of individual MLR analysis. An AUC value below 0.5 was considered no discrimination, 0.7≦AUC≦0.8 was considered acceptable discrimination, 0.8≦AUC≦0.9 was considered excellent discrimination, and 0.9≦AUC≦1.0 was considered outstanding discrimination [32]. Commercial statistical software, SPSS version 13.0, was used, and a two-tailed P < .05 was considered significant.

## Results

A total of 140 subjects were enrolled, and 112 subjects (60 men and 52 women) completed the study. The mean age, height, and body weight of the subjects were 69.6 ±10.3 years old (range, 45–89 years old), 158.1 ± 6.7 cm (range, 143–175 cm), and 61.2 ± 9.9 kg (range, 41–85 kg), respectively. Approximately half (50.8%) of all the subjects were right hemiplegic patients, and 88.4% of the subjects suffered stroke due to infarction.

Subjects were further divided into non-faller and faller groups depending on whether the subject experienced falls during the follow-up period. A total of 37 patients who experienced falls were classified into the faller group, and 75 subjects were classified into the non-faller group.

No significant differences were found in baseline measurements of age, gender, height, body weight, stroke affected side, stroke type, mental status, ambulation aids, or medications between faller and non-faller groups. However, physical and psychological assessments revealed that the faller group exhibited higher MAS and GDS and lower FIM and mFES scores compared to the non-faller group (Table 1). These physical and psychological assessments indicated that the faller group exhibited higher muscle tone, more severe depression, poor overall activity performance of daily life and lower confidence.

**Table 1:** Baseline measurements of the study subjects.

Groups Variables	All subjects (n = 112)	Non-faller (n = 75)	Faller (n = 37)	P value
Age	69.6 ± 10.3	69.9 ± 10.0	68.9 ± 10.8	0.629
Female (%)	52(46.4)	35(46.7)	17(46.0)	0.943
Height (cm)	158.1 ± 6.7	158.7 ± 6.7	157.1 ± 6.7	0.269
Weight (kg)	61.2 ± 9.9	61.5 ± 9.5	60.5 ± 10.7	0.635
Affected Side—right (%)	57(50.8)	38(50.7)	19(51.3)	0.946
Type—Infarction (%)	99(88.4)	67(89.3)	32(86.5)	0.853
MAS				
Elbow Flexor	0.7 ± 1.1	0.3 ± 0.8	1.4 ± 1.3	< 0.001
Quadriceps	0.6 ± 0.9	0.3 ± 0.7	1.1 ± 1.1	< 0.001
Gastrocnemius	0.6 ± 1.1	0.3 ± 0.7	1.3 ± 1.3	< 0.001
Soleus	0.6 ± 0.9	0.3 ± 0.7	1.1 ± 1.1	< 0.001
MMSE	21.8 ± 5.1	22.4 ± 4.8	20.5 ± 5.5	0.078
FIM				
Motor	79.6 ± 12.9	84.2 ± 10.0	71.1 ± 13.6	< 0.001
Cognition	29.9 ± 4.3	31.0 ± 3.8	28.0 ± 4.6	0.001
Total	109.5 ± 15.4	115.1 ± 12.3	99.1 ± 15.4	< 0.001
mFES	96.7 ± 33.8	108.5 ± 29.0	74.9 ± 31.3	< 0.001
GDS	4.5 ± 3.9	3.2 ± 3.3	7.1 ± 3.7	< 0.001
Ambulation Aids				
Independent walk (%)	35(31.3)	22(31.4)	13(31.0)	
Quadricane (%)	67(58.2)	42(60.0)	25(59.5)	0.947
Walker (%)	10(8.9)	6(8.6)	4(9.5)	
Medications				
Laxative (%)	66(0.59)	40(53.3)	26(70.3)	0.087
Benzodiazepines (%)	39(0.35)	23(30.7)	16(43.2)	0.189
Hypoglycemic (%)	12(0.11)	8(10.7)	4(10.8)	0.981
Antihypertensives (%)	45(0.40)	26(34.7)	19(51.4)	0.090

Values are % or mean ± SD.

MAS, Modified Ashworth Scale; MMSE, Mini-Mental State Exam; FIM, Functional Independence Measure; mFES, modified Fall Efficacy Scale; GDS, Geriatric Depression Scale.

We used an unbiased quantification using a computerized system to measure the balance and gait abilities in patients post-stroke to provide objective analyses. These computerized measurements were considered to be more objective tools than the traditional assessments [33].

The abilities of balance and gait were different between faller and non-faller groups. Computerized gait assessment revealed that the faller group exhibited slower walking velocity and fewer cadences compared to the non-faller group (P < .001) (Table 2). The temporal asymmetry ratios for ASY_ss, ASY_ds, and ASY_step were significantly greater (approximately twofold) in the faller group (P < .05). These results indicated that the faller group exhibited more severe asymmetry gait than the non-faller group.

**Table 2:** Comparison of balance and gait parameters in study subjects.

Groups Variables	All subjects (n = 112)	Non-faller (n = 75)	Faller (n = 37)	P value
Velocity (m/s)	0.48 ± 0.45	0.57 ± 0.51	0.28 ± 0.16	0.002
Cadence (steps/min)	87.75 ± 22.87	93.55 ± 19.26	76.00 ± 25.27	< 0.001
Asymmetry Ratio				
ASY_ss	0.23 ± 0.30	0.15 ± 0.15	0.39 ± 0.43	< 0.001
ASY_ds	0.26 ± 0.34	0.20 ± 0.21	0.38 ± 0.50	0.007
ASY_swing	0.32 ± 0.59	0.25 ± 0.64	0.45 ± 0.46	0.089
ASY_stance	0.07 ± 0.07	0.07 ± 0.07	0.08 ± 0.07	0.284
ASY_step	0.18 ± 0.31	0.11 ± 0.14	0.33 ± 0.47	< 0.001
Trajectory of COP				
COP_ml (mm)	3.43 ± 1.62	3.07 ± 1.59	4.11 ± 1.47	0.001
COP_ap (mm)	3.29 ± 1.41	3.17 ± 1.38	3.51 ± 1.45	0.229
COP_area (mm^2^)	37.62 ± 32.51	32.99 ± 30.94	46.51 ± 34.01	0.040

ASY_ss, asymmetry ratio of single support time; ASY_ds, asymmetry ratio of double support time; ASY_swing, asymmetry ratio of single swing time; ASY_stance, asymmetry ratio of stance time; ASY_step, asymmetry ratio of step time; COP, center of pressure; ml: medial-lateral; ap: anterior-posterior.

The faller group exhibited larger COP_area and greater COP_ml in computerized balance assessments (P < .01). These results demonstrated that the faller group exhibited worse postural sway in the mediolateral direction and area compared to the non-faller group. Therefore, the computerized gait and balance assessments may be used to accurately predict fall in the faller group.

Correlation analysis was also performed based on the results in Table 2 to determine the risk factors for predicting fall occurrence. Correlations between gait and balance variables were evaluated (Table 3). All parameters of the temporal asymmetry ratios negatively correlated with walking velocity and cadence. The COP_ml and COP_area exhibited a low-to-medium positive correlation with all parameters of the temporal asymmetry ratios. Therefore, the computer automatically selected ASY_ss and COP_ml to represent the gait and balance assessments, respectively, for further analysis.

**Table 3:** Correlation coefficients of balance and gait parameters (n = 112).

Variables	Cadence	Velocity	Trajectory of COP		
			ml	ap	area
Cadence (steps/min)	1.00	0.31†	−0.34^‡^	−0.16	−0.32^‡^
Velocity (m/s)	0.31†	1.00	−0.10	−0.02	−0.08
Asymmetry Ratio					
ASY_ss	−0.62^‡^	−0.26†	0.40^‡^	0.09	0.28†
ASY_ds	−0.50^‡^	−0.20*	0.23*	0.12	0.23*
ASY_swing	−0.54^‡^	−0.30†	0.49^‡^	0.20*	0.48^‡^
ASY_stance	−0.50^‡^	−0.23*	0.34^‡^	0.12	0.34^‡^
ASY_step	−0.61^‡^	−0.23*	0.32^‡^	0.14	0.29†
Trajectory of COP					
COP_ml (mm)	−0.34^‡^	−0.10	1.00	0.34^‡^	0.82^‡^
COP_ap (mm)	−0.16	−0.02	0.34^‡^	1.00	0.70^‡^
COP_area (mm^2^)	−0.32^‡^	−0.08	0.82^‡^	0.70^‡^	1.00

ASY_ss, asymmetry ratio of single support time; ASY_ds, asymmetry ratio of double support time; ASY_swing, asymmetry ratio of single swing time; ASY_stance, asymmetry ratio of stance time; ASY_step, asymmetry ratio of step time; COP, center of pressure; ml: Medial-Lateral; ap: Anterior-Posterior.

*P < .05

† *P* < .01

^‡^ *P* < .001.

Correlations between computerized gait and balance assessments and other physical or psychological assessments were further analyzed. The MAS of the gastrocnemius exhibited a low-to-medium positive correlation with COP_ml, ASY_ss, and GDS (Table 4). FIM also exhibited a medium negative correlation with MAS. This correlation analysis demonstrated that FIM negatively correlated with most of the physical and psychological assessments. The strength of the correlation was low-to-moderate between variables (Tables 3 and 4), but most correlations revealed significant differences. These results were used as variables for the subsequent MLR analysis.

**Table 4:** Correlation between predictors of risk of falls in stroke subjects (n = 112).

Variables	GDS	FIM	ASY_ss	COP_ml	MAS_gas
GDS	1.00	−0.48^‡^	0.17	0.42^‡^	0.39^‡^
FIM	−0.48^‡^	1.00	−0.46^‡^	−0.33^‡^	−0.34^‡^
ASY_ss	0.17	−0.46^‡^	1.00	0.39^‡^	0.20*
COP_ml	0.42^‡^	−0.33^‡^	0.39^‡^	1.00	0.26^‡^
MAS_gas	0.39^‡^	−0.34^‡^	0.20*	0.26^‡^	1.00

GDS, Geriatric Depression Score; FIM, Functional Independence Measure; ASY_ss, asymmetry ratio of single support; COP_ml, center of pressure in mediolateral direction; MAS_gas, Modified Ashworth Score of the gastrocnemius.

* *P* < .05

^‡^ *P* < .001.

The variables in Tables 3 and 4 were used for MLR analyses to determine the risk factors for predicting fall in stroke patients. Two models were subsequently generated. Table 5 shows that the significant predictors of fall occurrence (with P<0.05) in stroke patients were as follows in model I of the MLR analysis: (1) GDS (adjusted OR, 1.4; 95% CI, 1.2–1.8; P = .001); (2) gait asymmetry (ASY_ss) [aOR, 2.2; 95% CI, 1.2–3.8; P = .006]; and (3) spasticity of the gastrocnemius (aOR, 3.2; 95% CI, 1.4–7.3; P = .006). The sensitivity and specificity of this model were 82.6% and 86.5%, respectively, with a Youden index of 0.69. The model I analysis suggested that GDS, Gait Asymmetry (Single Support), and Spasticity (Gastrocnemius) were strong predictors for fall in stroke patients.

**Table 5:** Multivariate logistic regression for predictors of accidental falls.

Model	Factor	Coefficient (ß)	Adjusted odds ratio (95% CI)	P value
I	Geriatric Depression Scale	0.361	1.4 (1.2–1.8)^a^	0.001
	Gait Asymmetry (Single Support)	0.783	2.2 (1.2–3.8)^b^	0.006
	Spasticity (Gastrocnemius)	1.164	3.2 (1.4–7.3)^a^	0.006
Youden Index = 0.69; Sensitivity = 82.6%, Specificity = 86.5%				
II	Functional Independence Measure	−0.090	0.9 (0.9–1.0)^a^	0.002
	Gait Asymmetry (Single Support)	1.267	3.6 (1.4–9.2)^b^	0.009
	Postural Sway (Mediolateral)	0.518	1.7 (1.0–2.7)^a^	0.033
Youden Index = 0.53; Sensitivity = 76.9%, Specificity = 75.7%				

a: predicted change in odds for a unit increase in corresponding variables.

b: predicted change in odds for a standard deviation (SD = 0.3) in corresponding variable.

Notably, the commonly used measurement for regular functional assessment during stays in the rehabilitation unit, FIM, [34] was not automatically selected as one of the predictors after the MLR analysis in model I. This result may be attributed to the results that GDS exhibited the strongest negative correlation with FIM (-0.48) in the correlation analysis between risk factors in stroke subjects (Table 4). Therefore, GDS was excluded in another round of MLR analysis, and prediction model II was generated. Table 5 shows that the predictors of determining fall occurrence in model II included (1) FIM (aOR, 0.9; 95% CI, 0.9–1.0; P = .002), (2) gait asymmetry (ASY_ss) (aOR, 3.6; 95% CI, 1.4–9.2; P = .009), and (3) postural sway (mediolateral, COP_ml) (aOR, 1.7; 95% CI, 1.0–2.7; P = .033). Model II also exhibited relatively high sensitivity (76.9%) and specificity (75.7%) with a Youden index of 0.53, but the sensitivity and specificity were lower than model I.

**Fig 1:**
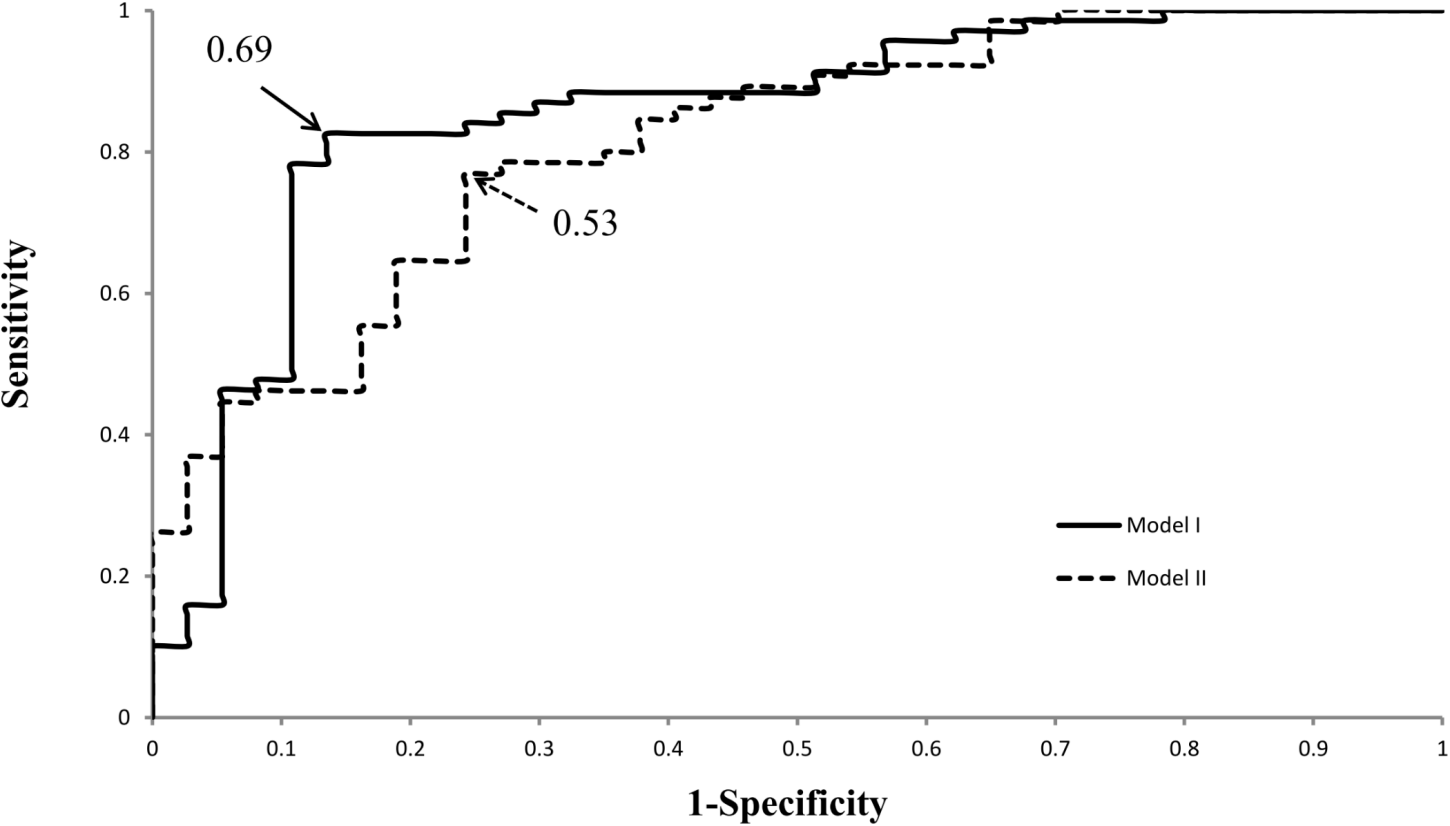
The ROC curves for predicting the occurrence of falls in stroke patients using models I and II. AUCs were 0.856 and 0.815, respectively. Arrowheads indicate the identified optimal cutoffs (Youden Index) for these prediction models (0.69 in model I and 0.53 in model II).

The ROC curves of the two models (Fig 1) for predicting falls in stroke patients were plotted to discriminate the two multivariate logistic models presented in Table 5. The ROC analysis revealed that model I (AUC value: 0.856) was better fitted than model II (AUC value 0.815). However, both models exhibited excellent fitness to predict fall occurrence in stroke patients with high sensitivity and specificity, with AUC values greater than 0.8 [32].

## Discussion

To our knowledge, this study is the first to include physical and psychological variables for determining the predictive risk factors of fall in stroke patients. The results underscore the significance of quantitative gait and balance assessments before discharge from rehabilitation units for predicting fall in stroke subjects by comparing the functional and baseline variables between the faller and non-faller groups of stroke subjects.

The faller group exhibited slower walking speed, asymmetrical gait, unstable balance, and lower functional performance than the non-faller group at baseline. Thirty-seven of the 112 enrolled subjects had at least one falling accident within 6 months after a stroke in this study (33% fall incidence).

Impaired gait symmetry, depression, and higher abnormal muscle tone were found in stroke patients who experienced falls. Prediction models for falls in stroke patients were developed using these physical and psychological parameters. The current findings provide sufficient information for predicting future falls, and early intervention strategies may be implemented to prevent falls in stroke patients.

### Assessment of falls

Previous studies reported that the “gold standard” for collecting information on falls (e.g., prospective collection with calendars or postcards, regular reminders, and follow-up telephone calls) was prone to errors (e.g., memory, forgetting to write diaries and ambiguous definitions of fall) [14]. To minimize these types of errors in this study, falls were recorded regularly by nurses during home visits 4, 12, and 24 weeks post-discharge and by subjects’ self-report. Recordings of fall history, environmental risk exam, and medical consultations were performed during the interviews with each subject. One advantage of the interview was to provide better interaction between subjects and research team workers. Therefore, subjects could fully understand the risk of falls and the ultimate goal of this study to prevent fall occurrence.

### Balance and gait performance

Poor postural balance was linked to increased fall risk in previous studies [11,35,36]. Mediolateral COP displacement during normal standing may be used as an indicator of accidental falls in the elderly because it was significantly associated with future falls [37]. The results of this study also demonstrated that postural sway in the mediolateral direction and area were greater in fallers compared to non-fallers.

A hemiparetic gait is described as slow and asymmetrical [38,39]. Walking velocity and cadence were lower in the faller group than the non-faller group in the present study. Gait speed is generally selected as the outcome measurement in clinical practice and a predictor of fall after a patient has a stroke, but gait speed is often confounded with balance, motor function, and endurance [40]. The current study adopted a quantitative gait analysis to help assess the risk of fall and further describe gait performance adequately.

A previous study reported that temporal gait symmetry measurement appeared to better reflect components related to weight shift, and it was superior to spatial symmetry ratios for identifying the risk of falls in impaired ambulators [38]. An “asymmetry ratio” was used to represent the level of temporal asymmetry (ASY_ss, ASY_ds, and ASY_step) in the present study, which was significantly different between fallers and non-fallers. An increase of one standard deviation in ASY_ss was associated with a 2.2 and 3.6 times higher fall risk in models I and II, respectively.

### Spasticity related to falls

An asymmetrical gait pattern caused by impaired balance and abnormal muscle tone is commonly seen in stroke patients. The present results demonstrated that the severity of spasticity in the upper and lower extremities was markedly higher in the faller group compared to the non-faller group. These findings are consistent with another study that also reported spasticity as a risk factor for predicting falls in chronic stroke patients. Motor control and functional status of stroke patients declined with increasing spasticity [13]. Logistic regression model I in this study also demonstrated that spasticity of the gastrocnemius was a predictor of fall in stroke patients. Another study found that the degree of spasticity of the affected ankle plantar flexors primarily influenced gait asymmetry [24]. A spastic gait in stroke patients diminished power generation, decreased hip and knee flexion during the swing phase, and reduced stability during the stance phase due to the affected hip flexors, knee extensors, and ankle plantar flexors [41]. The present study revealed that the risk of fall increased 3.2 times when the severity of spasticity in the gastrocnemius increased by one grade. The results also support that the combination of gait asymmetry and abnormal muscle tone may increase falls in the stroke population.

Previous studies reported that spasticity reached a peak within 1–3 months after a stroke [41–45]. Thirty-nine percent of patients who suffered a first stroke exhibited sustained spasticity after 12 months [19]. Therefore, the early detection of spasticity and improvement in motor dysfunction using specific interventions, such as stretching, splinting, electrical stimulation, and botulinum toxin injection, may be crucial to reduce accidental falls [46].

### Effects of functional performance

The Functional Independence Measure (FIM) is widely used to evaluate the performance of a patient’s daily activity to determine the level of a patient’s disability. All functional performance assessment results were significantly higher in non-fallers than fallers in our study. The fall risk decreased by 10% when the FIM score increased by one point. Previous studies also reported the significant correlation between FIM and fall occurrence [47–49]. A previous 10-year retrospective study also demonstrated the same correlation between the FIM score and fall risk [50], which is consistent with present results. However, the FIM score as a single variable may not be sufficient to accurately predict fall risk because falls generally resulted from multiple factors. The finding is also consistent with our MLR model, which enhanced the sensitivity and specificity of fall prediction.

### Effects of depression in stroke patients

Depressive symptoms are common in the acute phase after stroke, and symptoms are associated with the persistence of depression and mortality after 12 months [51]. The current MLR model in this study demonstrated that the risk of fall increased 1.4 times with a one-unit increase in GDS. Moreover, fall risk may cumulatively increase due to a high cognitive load if the patient also had multiple motor impairments and depression combined with gait asymmetry and spasticity.

### Fall prediction model

A bivariate correlation between risk predictors of accident fall in stroke subjects was performed to determine which variables to include in the MLR analysis. The results of bivariate correlation test revealed that the FIM and GDS exhibited the highest strength of negative correlation (Table 4). Therefore, two logistic regression models, including FIM or GDS, were developed in this study to determine the best fit of fall predictive factors. Notably, model I, which included GDS, gait asymmetry, and spasticity, exhibited slightly higher specificity, sensitivity, and Youden index than model II. Gait asymmetry, spasticity, and depression represented the functional, physical, and psychological domains of the subject’s impairments in function, respectively. Therefore, these results suggest that model I provides more comprehensive fall prediction than model II. ROC analysis further verified the discrimination of fitness of model I with a slightly greater AUC value (0.856) than model II (AUC value 0.815).

A previous study model with six predictors, including the Berg Balance score and functional performance, exhibited high predictive values (AUC = 0.712) in community stroke patients [52]. Both models in the present study used three predictors and demonstrated AUC values greater than 0.8. Several differences were observed between the two studies, including the race of enrolled subjects, time of assessment of falls post-stroke, and the selection of variables for MLR analysis. The present study included computerized gait asymmetry as a predictor in model II in addition to balance and functional predictors. The predictive values of model II reached AUC = 0.815 despite the inclusion of only three predictors. Computerized gait assessment was included in both models, and the computerized system may provide a more objective and accurate evaluation. Overall, these findings suggest that gait asymmetry is an important factor for the prediction of falls.

An earlier study also found that sideways fall was an independent risk factor (aOR, 2.5; 95% CI, 1.6–3.9) for hip fractures in the elderly, in which 20% of their population had a history of stroke. Therefore, preventing sideways fall may decrease the occurrence of hip fractures in the elderly [53]. Both prediction models in this study included gait asymmetry as one predictor of a fall. Model II included gait asymmetry and balance factors. Therefore, the fall prediction models, including computerized gait and balance assessments, may be used in stroke patients and the elderly for preventing possible falls.

### Study limitations

Several limitations may result from the present study design. First, the present study results cannot be extrapolated to all people with stroke, particularly patients at lower functional levels with walking disability or severe cognitive impairments. Second, the subjects were not separated into a construction data set and a validation data set to test the multivariate logistic regression function because of the small number of subjects enrolled.

## Conclusions

Multiple factors determine the risk of a fall in stroke patients, and a comprehensive assessment is needed to better understand the complex correlation between motor impairment, psychological factors, and the risk of falls in stroke patients.

The results of the present study revealed that the degree of depression, in addition to gait asymmetry and ankle spasticity, may play a crucial role in predicting a fall in stroke subjects. Therefore, more attention should be paid to emotional and social consequences in stroke patients in addition to regular intervention to improve physical function. The predictive factors determined in the present study provide additional prevention strategies for the healthcare team to prevent future falls in stroke patients after they return home.

## Supporting information

S1 Table ROC curves. (XLSX)

## Abbreviations

MAS: Modified Ashworth Scale
ASY: Asymmetry Ratio
COP: Center of Pressure
FIM: Functional Independence Measure
GDS: Geriatric Depression Scale
mFES: Modified Falls Efficacy Scale
MLR: Multivariate Logistic Regression

## References

[1] Stapleton T, Ashburn A, Stack E. A pilot study of attention deficits, balance control and falls in the subacute stage following stroke. Clin Rehabil. 2001;15: 437–444. doi: 10.1191/026921501678310243 11518445 10.1191/026921501678310243

[2] Hyndman D, Ashburn A, Stack E. Fall events among people with stroke living in the community: Circumstances of falls and characteristics of fallers. Arch Phys Med Rehabil. 2002;83: 165–170. 11833018 10.1053/apmr.2002.28030

[3] Jørgensen L, Engstad T, Jacobsen BK. Higher Incidence of Falls in Long-Term Stroke Survivors Than in Population Controls Depressive Symptoms Predict Falls After Stroke. Stroke. 2002;33: 542–547. 11823667 10.1161/hs0202.102375

[4] Hyndman D, Ashburn A. People with stroke living in the community: Attention deficits, balance, ADL ability and falls. Disabil Rehabil. 2003;25: 817–822. doi: 10.1080/0963828031000122221 12851091 10.1080/0963828031000122221

[5] Lamb SE, Ferrucci L, Volapto S, Fried LP, Guralnik JM. Risk Factors for Falling in Home-Dwelling Older Women With Stroke The Women’s Health and Aging Study. Stroke. 2003;34: 494–501. 12574566

[6] Kerse N, Parag V, Feigin VL, McNaughton H, Hackett ML, Bennett DA, et al Falls After Stroke Results From the Auckland Regional Community Stroke (ARCOS) Study, 2002 to 2003. Stroke. 2008;39: 1890–1893. doi: 10.1161/STROKEAHA.107.509885 18483413 10.1161/STROKEAHA.107.509885

[7] Tinetti ME, Speechley M, Ginter SF. Risk factors for falls among elderly persons living in the community. N Engl J Med. 1988;319: 1701–1707. doi: 10.1056/NEJM198812293192604 3205267 10.1056/NEJM198812293192604

[8] Thorbahn LDB, Newton RA. Use of the Berg Balance Test to predict falls in elderly persons. Phys Ther. 1996;76: 576–583. 8650273 10.1093/ptj/76.6.576

[9] Graafmans WC, Ooms ME, Hofstee HMA, Bezemer PD, Bouter LM, Lips P. Falls in the elderly: a prospective study of risk factors and risk profiles. Am J Epidemiol. 1996;143: 1129–1136. 8633602 10.1093/oxfordjournals.aje.a008690

[10] Schwesig R, Fischer D, Lauenroth A, Becker S, Leuchte S. Can falls be predicted with gait analytical and posturographic measurement systems? A prospective follow-up study in a nursing home population. Clin Rehabil. 2013;27: 183–190. doi: 10.1177/0269215512452880 22843355 10.1177/0269215512452880

[11] Maeda N, Kato J, Shimada T. Predicting the Probability for Fall Incidence in Stroke Patients Using the Berg Balance Scale. J Int Med Res. 2009;37: 697–704. doi: 10.1177/147323000903700313 19589253 10.1177/147323000903700313

[12] Jalayondeja C, Sullivan PE, Pichaiyongwongdee S. Six-month prospective study of fall risk factors identification in patients post-stroke. Geriatr Gerontol Int. 2014;14: 778–785. doi: 10.1111/ggi.12164 24666687 10.1111/ggi.12164

[13] Soyuer F, Öztürk A. The effect of spasticity, sense and walking aids in falls of people after chronic stroke. Disabil Rehabil. 2007;29: 679–687. doi: 10.1080/09638280600925860 17453990 10.1080/09638280600925860

[14] Mansfield A, Wong JS, McIlroy WE, Biasin L, Brunton K, Bayley M, et al Do measures of reactive balance control predict falls in people with stroke returning to the community? Physiotherapy. 10.1016/j.physio.2015.01.009 26050134

[15] Alzahrani MA, Dean CM, Ada L, Dorsch S, Canning CG. Mood and Balance are Associated with Free-Living Physical Activity of People after Stroke Residing in the community. Stroke Res Treat. 2012;2012: 1–8. 10.1155/2012/470648 PMC3195499 22013550

[16] Paolucci S. Epidemiology and treatment of post-stroke depression. Neuropsychiatr Dis Treat. 2008;4: 145–154. 18728805 10.2147/ndt.s2017 PMC2515899

[17] Weerdesteyn V, de Niet M, van Duijnhoven HJ, CH A, Geurts MD. Falls in individuals with stroke. differences. 2008;33: 36. 19235120

[18] Persson CU, Hansson P-O, Sunnerhagen KS. Clinical Tests Performed in Acute Stroke Identify the Risk of Falling During the First Year: Postural Stroke Study in Gothenburg (Postgot). J Rehabil Med. 2011;43: 348–353. doi: 10.2340/16501977-0677 21267528 10.2340/16501977-0677

[19] Watkins CL, Leathley MJ, Gregson JM, Moore AP, Smith TL, Sharma AK. Prevalence of spasticity post stroke. Clin Rehabil. 2002;16: 515–522. doi: 10.1191/0269215502cr512oa 12194622 10.1191/0269215502cr512oa

[20] Granger CV, Hamilton BB, Linacre JM, Heinemann AW, Wright BD. Performance profiles of the functional independence measure. Am J Phys Med Rehabil. 1993;72: 84–89. 8476548 10.1097/00002060-199304000-00005

[21] Teasell R, McRae M, Foley N, Bhardwaj A. The incidence and consequences of falls in stroke patients during inpatient rehabilitation: Factors associated with high risk. Arch Phys Med Rehabil. 2002;83: 329–333. 11887112 10.1053/apmr.2002.29623

[22] Dusing SC, Thorpe DE. A normative sample of temporal and spatial gait parameters in children using the GAITRite® electronic walkway. Gait Posture. 2007;25: 135–139. doi: 10.1016/j.gaitpost.2006.06.003 16875823 10.1016/j.gaitpost.2006.06.003

[23] Kim CM, Eng JJ. Symmetry in vertical ground reaction force is accompanied by symmetry in temporal but not distance variables of gait in persons with stroke. Gait Posture. 2003;18: 23–28. 12855297 10.1016/s0966-6362(02)00122-4

[24] Hsu A-L, Tang P-F, Jan M-H. Analysis of impairments influencing gait velocity and asymmetry of hemiplegic patients after mild to moderate stroke. Arch Phys Med Rehabil. 2003;84: 1185–1193. 12917858 10.1016/s0003-9993(03)00030-3

[25] Swanenburg J, Bruin ED de, Favero K, Uebelhart D, Mulder T. The reliability of postural balance measures in single and dual tasking in elderly fallers and non-fallers. BMC Musculoskelet Disord. 2008;9: 162 doi: 10.1186/1471-2474-9-162 19068125 10.1186/1471-2474-9-162 PMC2614424

[26] Garland SJ, Ivanova TD, Mochizuki G. Recovery of Standing Balance and Health-Related Quality of Life After Mild or Moderately Severe Stroke. Arch Phys Med Rehabil. 2007;88: 218–227. doi: 10.1016/j.apmr.2006.11.023 17270520 10.1016/j.apmr.2006.11.023

[27] Folstein MF, Folstein SE, McHugh PR. “Mini-mental state”: A practical method for grading the cognitive state of patients for the clinician. J Psychiatr Res. 1975;12: 189–198. 1202204 10.1016/0022-3956(75)90026-6

[28] Chan AC-M. Clinical validation of the geriatric depression scale (GDS) Chinese version. J Aging Health. 1996;8: 238–253. doi: 10.1177/089826439600800205 10160560 10.1177/089826439600800205

[29] Hill KD, Schwarz JA, Kalogeropoulos AJ, Gibson SJ. Fear of falling revisited. Arch Phys Med Rehabil. 1996;77: 1025–1029. 8857881 10.1016/s0003-9993(96)90063-5

[30] Bewick V, Cheek L, Ball J. Statistics review 13: Receiver operating characteristic curves. Crit Care. 2004;8: 508 doi: 10.1186/cc3000 15566624 10.1186/cc3000 PMC1065080

[31] Akobeng AK. Understanding diagnostic tests 3: receiver operating characteristic curves. Acta Pædiatrica. 2007;96: 644–647. doi: 10.1111/j.1651-2227.2006.00178.x 17376185 10.1111/j.1651-2227.2006.00178.x

[32] Hosmer DW, Lemeshow S. Assessing the Fit of the Model. Applied Logistic Regression. John Wiley & Sons, Inc.; 2000 pp. 143–202.

[33] Wong AMK, Chen C-L, Chen CPC, Chou S-W, Chung C-Y, Chen MJL. Clinical Effects of Botulinum Toxin A and Phenol Block on Gait in Children with Cerebral Palsy. Am J Phys Med Rehabil. 2004;83: 284–291. 15024330 10.1097/01.phm.0000118038.02326.ca

[34] Ring H, Feder M, Schwartz J, Samuels G. Functional measures of first stroke in patient rehabilitation: The use of the FIM total score with a clinical rationale. Arch Phys Med Rehabil. 1997;78: 630–5. 9196471 10.1016/s0003-9993(97)90429-9

[35] Harris JE, Eng JJ, Marigold DS, Tokuno CD, Louis CL. Relationship of Balance and Mobility to Fall Incidence in People With Chronic Stroke. Phys Ther. 2005;85: 150–158. 15679466

[36] Belgen B, Beninato M, Sullivan PE, Narielwalla K. The Association of Balance Capacity and Falls Self-Efficacy With History of Falling in Community-Dwelling People With Chronic Stroke. Arch Phys Med Rehabil. 2006;87: 554–561. doi: 10.1016/j.apmr.2005.12.027 16571397 10.1016/j.apmr.2005.12.027

[37] Piirtola M, Era P. Force platform measurements as predictors of falls among older people–a review. Gerontology. 2006;52: 1–16. doi: 10.1159/000089820 16439819 10.1159/000089820

[38] Patterson KK, Parafianowicz I, Danells CJ, Closson V, Verrier MC, Staines WR, et al Gait Asymmetry in Community-Ambulating Stroke Survivors. Arch Phys Med Rehabil. 2008;89: 304–310. doi: 10.1016/j.apmr.2007.08.142 18226655 10.1016/j.apmr.2007.08.142

[39] Patterson KK, Gage WH, Brooks D, Black SE, McIlroy WE. Changes in Gait Symmetry and Velocity After Stroke: A Cross-Sectional Study From Weeks to Years After Stroke. Neurorehabil Neural Repair. 2010; 10.1177/1545968310372091 20841442

[40] van de Port IG, Kwakkel G, Lindeman E. Community ambulation in patients with chronic stroke: how is it related to gait speed? J Rehabil Med. 2008;40: 23–27. doi: 10.2340/16501977-0114 18176733 10.2340/16501977-0114

[41] Fellows SJ, Ross HF, Thilmann AF. The limitations of the tendon jerk as a marker of pathological stretch reflex activity in human spasticity. J Neurol Neurosurg Psychiatry. 1993;56: 531–537. 8505646 10.1136/jnnp.56.5.531 PMC1015014

[42] Crone C, Johnsen LL, Biering-Sørensen F, Nielsen JB. Appearance of reciprocal facilitation of ankle extensors from ankle flexors in patients with stroke or spinal cord injury. Brain. 2003;126: 495–507. 12538415 10.1093/brain/awg036

[43] Ju M-S, Chen J-JJ, Lee H-M, Lin T-S, Lin C, Huang Y-Z. Time-course analysis of stretch reflexes in hemiparetic subjects using an on-line spasticity measurement system. J Electromyogr Kinesiol. 2000;10: 1–14. 10659445 10.1016/s1050-6411(99)00018-8

[44] O’Dwyer NJ, Ada L, Neilson PD. Spasticity and muscle contracture following stroke. Brain. 1996;119: 1737–1750. 8931594 10.1093/brain/119.5.1737

[45] Thilmann AF, Fellows SJ, Garms E, others. The mechanism of spastic muscle hypertonus. Brain. 1991;114: 233–244. 1998884

[46] Johnson CA, Burridge JH, Strike PW, Wood DE, Swain ID. The effect of combined use of botulinum toxin type A and functional electric stimulation in the treatment of spastic drop foot after stroke: a preliminary investigation1. Arch Phys Med Rehabil. 2004;85: 902–909. 15179643 10.1016/j.apmr.2003.08.081

[47] Saverino A, Benevolo E, Ottonello M, Zsirai E, Sessarego P. Falls in a rehabilitation setting: functional independence and fall risk. Eur Medicophysica. 2006;42: 179–184. 17039213

[48] Lee JE, Stokic DS. Risk factors for falls during inpatient rehabilitation. Am J Phys Med Rehabil. 2008;87: 341–353. doi: 10.1097/PHM.0b013e31816ddc01 18427218 10.1097/PHM.0b013e31816ddc01

[49] Suzuki T, Sonoda S, Misawa K, Saitoh E, Shimizu Y, Kotake T. Incidence and Consequence of Falls in Inpatient Rehabilitation of Stroke Patients. Exp Aging Res. 2005;31: 457–469. doi: 10.1080/03610730500206881 16147463 10.1080/03610730500206881

[50] Petitpierre NJ, Trombetti A, Carroll I, Michel J-P, Herrmann FR. The FIM® instrument to identify patients at risk of falling in geriatric wards: a 10-year retrospective study. Age Ageing. 2010;39: 326–331. doi: 10.1093/ageing/afq010 20172852 10.1093/ageing/afq010

[51] Kouwenhoven SE, Kirkevold M, Engedal K, Kim HS. Depression in acute stroke: prevalence, dominant symptoms and associated factors. A systematic literature review. Disabil Rehabil. 2011;33: 539–556. doi: 10.3109/09638288.2010.505997 20690858 10.3109/09638288.2010.505997

[52] Ashburn A, Hyndman D, Pickering R, Yardley L, Harris S. Predicting people with stroke at risk of falls. Age Ageing. 2008;37: 270–276. doi: 10.1093/ageing/afn066 18456791 10.1093/ageing/afn066

[53] Wei TS, Hu CH, Wang SH, Hwang, K. L. Fall characterictics, functional mobility and bone mineral density as risk factors of hip fracture in the community-dwelling ambulatory elderly. Osteoporos Int. 2001;12: 1050–1055. 11846332 10.1007/pl00004184

